# On some properties of the generalized Mittag-Leffler function

**DOI:** 10.1186/2193-1801-2-337

**Published:** 2013-07-23

**Authors:** Mumtaz Ahmad Khan, Shakeel Ahmed

**Affiliations:** Department of Applied Mathematics, Faculty of Engineering, Aligarh Muslim University, Aligarh, 202002 India

**Keywords:** Mittag-Leffler function, Generalized hypergeometric function, Fox’s H function

## Abstract

**Abstract:**

This paper deals with the study of a generalized function of Mittag-Leffler type. Various properties including usual differentiation and integration, Euler(Beta) transforms, Laplace transforms, Whittaker transforms, generalized hypergeometric series form with their several special cases are obtained and relationship with Wright hypergeometric function and Laguerre polynomials is also established.

**2000 Mathematics Subject Classification:**

33C45, 47G20, 26A33.

## Introduction

In 1903, the Swedish mathematician Gosta Mittag-Leffler (1903) introduced the function 1.1

where *z* is a complex variable and *Γ* is a Gamma function *α* ≥ 0. The Mittag-Leffler function is a direct generalisation of exponential function to which it reduces for *α* = 1. For 0 < *α* < 1 it interpolates between the pure exponential and hypergeometric function  Its importance is realized during the last two decades due to its involvement in the problems of physics, chemistry, biology, engineering and applied sciences. Mittag-Leffler function naturally occurs as the solution of fractional order differential or fractional order integral equation.

The generalisation of *E*_*α*_(*z*) was studied by Wiman (1905) in 1905 and he defined the function as 1.2

which is known as Wiman function.

In 1971, Prabhakar (1971) introduced the function  in the form of 1.3

where (*γ*)_*n*_ is the Pochhammer symbol (Rainville (1960)) 

In 2007, Shukla and Prajapati (2007) introduced the function  which is defined for ; Re(*α*) > 0,Re(*β*) > 0,Re(*γ*) > 0 and  as 1.4

In 2009, Tariq O. Salim (2009) introduced the function the function  which is defined for  as 1.5

In 2012, a new generalization of Mittag-Leffler function was defined by Salim (2012) as 1.6

where  min (Re(*α*), Re(*β*), Re(*γ*), Re(*δ*)) > 0

In this paper a new definition of generalized Mittag-Leffler function is investigated and defined as 1.7

where 1.8

Further the generalization of definition (1.7) is investigated and defined as follows 1.9

where, 1.10

and 1.11

The definition (1.9) is a generalization of all above functions defined by (1.1)-(1.7).

- Setting *μ* = *ν*, *ρ* = *σ*, it reduces to Eq. (1.6) defined by Salim (2012), in addition of that if *p* = 1, it reduces to Eq. (1.7).
- Setting *μ* = *ν*, *ρ*, = *σ* and *p* = *q* = 1, it reduces to Eq. (1.5) defined by Salim (2009).
- Setting *μ* = *ν*, *ρ* = *σ* and *p* = *δ* = 1, it reduces to Eq. (1.4) defined by Shukla and Prajapati (2007), in addition of that if *q* = 1, then we get Eq. (1.3) defined by Prabhakar (1971).
- Setting *μ* = *ν*, *ρ* = *σ* and *p* = *q* = *δ* = 1, it reduces to Eq. (1.2) defined by Wiman (1905), moreover if *β* = 1, Mittag-Leffler function *E*_*α*_(*z*) will be the result.

Throughout this investigation, we need the following well-known facts to study the various properties and relation formulas of the function .

- Beta(Euler) transforms (Sneddon (1979)) of the function *f*(*z*) is defined as 1.12
- Laplace transforms (Sneddon (1979)) of the function *f*(*z*) is defined as 1.13
- Mellin- transforms of the function *f*(*z*) is defined as 1.14
- and the inverse Mellin-transform is given by 1.15
- Whittaker transform (Whittaker and Watson (1962)) 1.16
- where  and *W*_*λ*,*μ*_(*t*) is the Whittaker confluent hypergeometric function.
- The generalized hypergeometric function (Rainville (1960)) is defined as 1.17
- Wright generalized hypergeometric function (Srivastava and Manocha (1984)) is defined as 1.18
- Fox’s H-function (Saigo and Kilbas (1998)) is given as 1.19
- Generalized Laguerre polynomials (Rainville (1960)). These are also known as Sonine polynomials and are defined as 1.20
- Incomplete Gamma function (Rainville (1960)). This is denoted by *γ*(*α*, *z*) and is defined by 1.21

## Basic properties of the function

As a consequence of definitions (1.1)-(1.9) the following results hold:

### Theorem 2.1.

If , Re(*α*) > 0, Re(*β*) > 0, Re(*γ*) > 0, Re(*δ*) > 0, Re(*μ*) > 0, Re(*ν*) > 0, Re(*ρ*) > 0, Re(*σ*) > 0 *and p*, *q* > 0 *andq* ≤ Re(*α*) + *p*, then2.1.12.1.22.1.3

In particular, 2.1.42.1.52.1.6

### Proof.



which is (2.1.1).

The proof of (2.1.2) can easily be followed from the definition (1.9). Now 

which proves (2.1.3). □

- Substituting *μ* = *ν*, *ρ* = *σand p* = 1 in (2.1.1) immediately leads to (2.1.4).
- Substituting *μ* = *ν*, *ρ* = *σand p* = 1 in (2.1.2) immediately leads to (2.1.5).
- Putting *μ* = *ν*,*ρ* = *σand p* = 1 in (2.1.3) immediately leads to (2.1.6).

### Theorem 2.2.

If , Re(*α*) > 0, Re(*β*) > 0, Re(*γ*) > 0, Re(*δ*) > 0, Re(*μ*) > 0, Re(*ν*) > 0, Re(*ρ*) > 0, Re(*σ*) > 0, Re(w) > 0; *andq* ≤ Re(*α*) + p then for 2.2.12.2.2

In particular, 2.2.32.2.4

### Proof.

From (1.9), 

which is the proof of (2.2.1).

Again using (1.9) and term by term differentiation under the sign summation(which is possible in accordance with the uniform convergence of the series (1.9) in any compact set ), we have 

which is the proof of (2.2.2). □

- Setting *μ* = *ρ*,*ν* = *σ*, in (2.2.1), we get (2.2.3).
- Setting *μ* = *ρ*, *ν* = *σ*, in (2.2.2), we get (2.2.4).

### Theorem 2.3.

If , with  relatively prime; and q < Re(*α* + p), then2.3.12.3.2

### Proof.



which proves (2.3.1). □

### Corollary 2.3.

For *μ* = *ν*, *ρ* = *σ*, *δ* = *p* = 1, (2.3.1) reduces to the known result of Shukla and Prajapati Shukla and Prajapati (2007) (2.3.1).

### Remark 2.3.

Setting *μ* = *ν*, *ρ* = *σand p* = 1 in (2.3.1), we get (2.3.2).

**Special Properties:** Setting putting *μ* = *ν*, *ρ* = *σand p* = *q* = *δ* = 1 in (2.3.1), we have2.3.3

For *β* = *γ* = *δ* = *q* = 1 in (2.3.2), we have 2.3.4

### Theorem 2.4.

If  then2.4.12.4.22.4.3

In particular, 2.4.42.4.52.4.62.4.72.4.8

### Proof.



which proves (2.4.1).

Now change the variable from *s* to  Then the L.H.S. of (2.4.2) becomes 

which proves (2.4.2).

Now 

which proves (2.4.3).

Putting *q* = *δ* = 1 and *γ* = *q* = *δ* = 1 in (2.4.1) and (2.4.3) yields (2.4.4) and (2.4.5) respectively. □

## Generalized hypergeometric function representation of 

Using (1.9) with  and , we have 

where *Δ*(*l*;*μ*) is a *l*-tupple ; *Δ*(*q*;*γ*) is a *q*-tupple ; *Δ*(*k*, *β*) is a *k*-tupple  and so on, which is the required hypergeometric representation.

Convergence criterion of generalized Mittag-leffler function _*q*+*l*+1_*F*_*k*+*p*+*m*_: **(i)** If *q* + *l* + 1 ≤ *k* + *p* + *m*, the function _*q*+*l*+1_*F*_*k*+*p*+*m*_ converges for all finite z. **(ii)** If *q* + *l* + 1 = *k* + *p* + *m* + 1, the function _*q*+*l*+1_*F*_*k*+*p*+*m*_ converges for |*z*| < 1 and diverges for |*z*| > 1**(iii)** If *q* + *l* + 1 > *k* + *p* + *m* + 1, the function _*q*+1+1_*F*_*k*+*p*+*m*+1_ is divergent for |*z*| ≠ 0**(iv)** If *q* + *l* + 1 = *k* + *p* + *m* + 1, the function _*q*+*l*+1_*F*_*k*+*p*+*m*+1_ is absolutely convergent on the circle for |*z*| = 1, if 

## Integral transforms of 

In this section we discuss some useful integral transforms like Euler transform, laplace transform and Whittaker transform of 

### Theorem 4.1.

Mellin-Barnes integral representation of 

Let (1.9) and (1.10) be satified and  and *q* < *Re*(*α*) + *p*. Then the function  is represented by Mellin-Barnes integral as:4.1.1

where | arg(*z*)| < 1; the contour of integration beginning at −*i∞* and ending at +*i∞*, and indented to separate the poles of the integrand at  (to the left) from those at  (to the right).

### Proof.

We shall evaluate the integral on R.H.S. of (4.1.1) as the sum of the residues at the poles *s* = 0, − 1, − 2, …, we have 

which completes the proof. □

### Remark 4.1.

Setting *μ* = *ρ*, *ν* = *σand p* = 1, we get the Melin Barne’s integral of the function 

### Theorem 4.2.

**(Mellin transform) of**4.2.1

### Proof.

From Theorem 4.1, we have 

where 

is in the form of inverse Mellin-Transform (1.15). So applying the Mellin-transform (1.14) yields directly the required result. □

### Theorem 4.3.

(Euler(Beta)transform) of4.3.1

### Proof.



from which the result follows. □

### Corollary 4.3.

4.3.2

**Special properties: (i)** For *q* = 1, (4.3.2) reduces to Tariq OSalim (2009)(4.1). 4.3.3

**(ii)** For *δ* = *q* = 1 in (4.3.2), we have 4.3.4

If *a* = *β*, *α* = *σ*, then (4.3.2) reduces to 4.3.5

Putting *α* = *β* = *γ* = *δ* = *q* = 1 in (4.3.2), we have 4.3.6

### Theorem 4.4.

**(Laplace transform)**4.4.1

### Proof.



from which the result follows. □

### Corollary 4.4.

4.4.2

### Remark 4.4.

For *q* = 1, (4.4.2) reduces to Tariq O Salim (2009)(4.2).

### Theorem 4.5.

**(Whittaker transform)**4.5.1

### Proof.

Substituting *ϕt* = *v* in L.H.S. of Theorem 4.5, we have 

from which the result follows. □

### Corollary 4.5.

4.5.2

**Special properties :(i)** Putting *q* = *δ* = 1 in (4.5.2), we have 4.5.3

**(ii)** For *q* = *γ* = *δ* = 1 in (4.5.2), we have 4.5.4

**(iii)** Now putting *q* = *β* = *α* = *γ* = *δ* = 1 in (4.5.2), we have 4.5.5

## Relationship with some known special functions

### Relationship with Wright hypergeometric function

If the condition (1.10) be satisfied, then (1.9) can be written as 5.1.1

### Relationship with Fox H-function

Using (4.1.1), we have from 5.2.1

### Relationship with generalized Laguerre polynomials

Putting *α* = *k*, *β* = *μ* + 1, *γ* = − *m*, *q* ∈ *N* with *q*|*m* and replacing z by *z*^*k*^ in (1.6), we get 

where  is a generalization of (given by Shukla *et al*^2007^).

Note that  is a polynomial of degree  in *z*^*k*^.

Further for , where  is a generalized Laguerre polynomial. So that 

which is the required relationship.
